# Safety of SGLT2 inhibitors versus DPP-4 inhibitors in super-elderly patients (≥ 80 years) with type 2 diabetes: a propensity score-matched cohort study

**DOI:** 10.1186/s40842-026-00305-4

**Published:** 2026-06-29

**Authors:** Shankar Biswas, Yashasvi Srivastava, Ahmed Wahba, Bushra Amer, Ayman Hamadttu

**Affiliations:** 1 Department of Internal Medicine, Ivano-Frankivsk National Medical University, Ivano-Frankivsk, Ukraine; 2 Faculty of Medicine, Port Said University, Port Said, Egypt; 3 Department of Internal Medicine, Berkshire Medical Center, Pittsfield, USA; 4 Sudan University of Science and Technology, Khartoum, Sudan

**Keywords:** SGLT2 inhibitors, DPP-4 inhibitors, Type 2 diabetes, Elderly, Drug safety, Falls, Fractures, Mortality

## Abstract

**Background:**

Sodium-glucose cotransporter-2 inhibitors (SGLT2i) provide cardiovascular and renal benefits in type 2 diabetes, yet safety data in super-elderly patients (≥ 80 years) remain limited. We compared the safety of SGLT2i versus dipeptidyl peptidase-4 inhibitors (DPP-4i) in this vulnerable population.

**Methods:**

We conducted a retrospective, new-user, active-comparator cohort study using the TriNetX Global Collaborative Network (176 healthcare organizations). Patients aged ≥ 80 years at treatment initiation with type 2 diabetes newly prescribed SGLT2i or DPP-4i were matched 1:1 using propensity scores incorporating demographics, comorbidities, frailty proxies, and concomitant medications. Primary safety outcomes included falls, hip fracture, acute kidney injury, urinary tract infection, genital candidiasis, hypoglycemia, volume depletion, hypotension, and syncope. Secondary outcomes included stroke, myocardial infarction, heart failure hospitalization, and all-cause mortality. Subgroup analyses assessed falls risk among insulin, benzodiazepine, and loop diuretic users.

**Results:**

After matching, 65,119 patients remained in each cohort (mean age 82.2 ± 2.3 years; 48.7% female). Over a mean follow-up of 269 days, SGLT2i was associated with significantly lower risks of falls (HR 0.90; 95% CI 0.85–0.95), hip fracture (HR 0.78; 0.69–0.89), acute kidney injury (HR 0.82; 0.78–0.85), urinary tract infection (HR 0.79; 0.75–0.83), hypoglycemia (HR 0.74; 0.67–0.82), volume depletion (HR 0.91; 0.86–0.97), stroke (HR 0.88; 0.82–0.95), and all-cause mortality (HR 0.73; 0.70–0.76; all *p* < 0.05). Genital candidiasis risk was higher with SGLT2i (HR 1.70; 1.50–1.93; *p* < 0.001), as was heart failure hospitalization (HR 1.07; 1.02–1.12; *p* = 0.007). Hypotension, syncope, and myocardial infarction did not differ significantly. The falls reduction was consistent across subgroups using insulin, benzodiazepines, and loop diuretics. Sensitivity analysis with fixed 250-day follow-up confirmed all primary findings.

**Conclusions:**

In super-elderly patients with type 2 diabetes, SGLT2i demonstrated a favorable safety profile compared to DPP-4i, with significantly lower risks of falls (10%), hip fracture (22%), acute kidney injury (18%), hypoglycemia (26%), and mortality (27%). Safety concerns included increased genital candidiasis (70%) and modestly higher heart failure hospitalization (7%). These findings support SGLT2i use in appropriately selected super-elderly patients.

**Clinical trial number:**

Not applicable.

**Supplementary Information:**

The online version contains supplementary material available at 10.1186/s40842-026-00305-4.

## Background

Type 2 diabetes mellitus (T2DM) in the super-elderly (aged ≥ 80 years) represents one of the fastest-growing segments of the diabetic population. In the United States, diabetes prevalence reaches 21.7% in men and 15.6% in women aged ≥ 80 years, with this group showing the highest relative rate of increase (2.35-fold) over recent decades [1]. Globally, diabetes prevalence in those aged ≥ 65 years stands at 135.6 million (95% CI: 107.6–170.6 million), projected to exceed 276 million by 2045 [2]. Managing T2DM in this population presents unique challenges, including frailty, polypharmacy, cognitive impairment, shortened life expectancy, and heightened susceptibility to treatment-related adverse effects, particularly hypoglycemia [3].

Sodium-glucose cotransporter-2 inhibitors (SGLT2i) have transformed diabetes management through landmark cardiovascular outcomes trials demonstrating reductions in major adverse cardiovascular events, heart failure hospitalizations, and chronic kidney disease progression [4–6]. Empagliflozin reduced cardiovascular mortality with consistent benefits in patients aged ≥ 75 years (HR 0.55; 95% CI 0.32–0.94) [4], while dapagliflozin reduced cardiovascular death and heart failure hospitalization in the largest SGLT2i trial to date [5]. Canagliflozin established similar cardiovascular benefits but raised fracture concerns (HR 1.26; 95% CI 1.04–1.52), particularly relevant for elderly patients [6].

Despite these efficacy data, super-elderly patients have been markedly underrepresented in pivotal trials. Patients aged ≥ 75 years comprised only 10–11% of EMPA-REG OUTCOME [4] and 6.4% of DECLARE-TIMI 58 [5], with no trial reporting outcomes specifically for those aged ≥ 80 years. This reflects a systemic issue: across 440 diabetes trials, 65.7% excluded participants using upper age limits; consequently, adults aged ≥ 75 years comprised merely 1% of trial populations [7].

Current guidelines recommend SGLT2i for patients with established cardiovascular or renal comorbidities but acknowledge limited evidence in the oldest old [8, 9], providing no specific guidance for patients aged ≥ 80 years. Safety concerns - including volume depletion, acute kidney injury, and genital mycotic infections - have further contributed to prescribing hesitancy, despite a large Medicare study (*n* = 137,667) demonstrating no increased fracture risk and notably lower fall risk with SGLT2i versus DPP-4i (HR 0.82; 95% CI 0.77–0.87) [10]. Empagliflozin’s prescribing information explicitly discourages initiation in those aged ≥ 85 years [11].

DPP-4i have consequently become the default choice for elderly patients given their favorable safety profile [12], yet they lack the cardioprotective and renoprotective benefits of SGLT2i a clinically significant trade-off when comorbidities are present. No head-to-head randomized trials have directly compared these drug classes in any elderly cohort [13].

To address this evidence gap, we conducted a large propensity score-matched retrospective cohort study using the TriNetX Global Collaborative Network (176 Healthcare organizations) [14], comparing SGLT2i versus DPP-4i in patients aged ≥ 80 years across geriatric-relevant safety outcomes - falls, hip fractures, acute kidney injury, volume depletion, hypoglycemia, urinary tract infections, genital candidiasis, hypotension and syncope - alongside cardiovascular effectiveness endpoints and all-cause mortality.

## Methods

### Study design and data source

We conducted a retrospective, new-user, active-comparator cohort study using the TriNetX Global Collaborative Network, a federated health research network aggregating real-time electronic health record data from 176 participating healthcare organizations [14]. The analysis was conducted in March 2026, with 171 healthcare organizations online at the time of query execution. This study was exempt from institutional review board approval as TriNetX provides only de-identified, aggregate-level data compliant with the Health Insurance Portability and Accountability Act (HIPAA).

### Study population

The study population comprised patients aged ≥ 80 years at treatment initiation with type 2 diabetes mellitus (ICD-10: E11) who were newly prescribed either a sodium-glucose cotransporter-2 inhibitor (SGLT2i) or a dipeptidyl peptidase-4 inhibitor (DPP-4i). We employed a new-user design requiring no prior exposure to either medication class during the 1 year before the index date.

The SGLT2i cohort included patients receiving empagliflozin, dapagliflozin, canagliflozin, or ertugliflozin at the index date, with age ≥ 80 years at the time of first prescription. The DPP-4i cohort included patients receiving sitagliptin, saxagliptin, linagliptin, or alogliptin at the index date, with age ≥ 80 years at the time of first prescription.

Patients were excluded if they had end-stage renal disease (ICD-10: N18.6), dependence on renal dialysis (ICD-10: Z99.2), kidney transplant status (ICD-10: Z94.0), hemodialysis procedures (CPT: 90935, 90937), other dialysis procedures (CPT: 90945), or prior use of medications from either drug class within 1 year before the index date.

### Frailty assessment

Validated frailty scales are not directly captured in TriNetX; therefore, we used established electronic health record-based frailty proxies as matching variables. These included vascular dementia (ICD-10: F01), dementia in other diseases classified elsewhere (ICD-10: F02), unspecified dementia (ICD-10: F03), Alzheimer’s disease (ICD-10: G30), mild cognitive impairment (ICD-10: G31.84), sarcopenia (ICD-10: M62.84), muscle weakness (ICD-10: M62.81), abnormality of gait and mobility (ICD-10: R26), history of falls (ICD-10: W00-W19), prior femoral fracture (ICD-10: S72), osteoporosis (ICD-10: M80, M81), adult failure to thrive (ICD-10: R62.7), cachexia (ICD-10: R64), and malnutrition (ICD-10: E40-E46). These proxies have been validated as components of claims-based frailty indices and are associated with adverse outcomes in elderly populations [15, 16].

### Baseline renal function

Baseline renal function was characterized using ICD-10 coded chronic kidney disease stages. Patients with CKD (ICD-10: N18), including stages 3 (N18.3), 4 (N18.4), and 5 non-dialysis, were identified. Patients on dialysis or with ESRD requiring renal replacement therapy were excluded as described above.

### Concomitant medications

Baseline concomitant medications with known effects on falls, fractures, or hypoglycemia were assessed in the 12 months prior to the index date and included as matching variables. Diabetes medications included insulin, metformin, sulfonylureas (glipizide, glimepiride, glyburide), and GLP-1 receptor agonists (semaglutide, liraglutide, dulaglutide, exenatide). Cardiovascular medications included beta-blockers, loop diuretics, thiazide diuretics, potassium-sparing diuretics, ACE inhibitors, angiotensin receptor blockers, and calcium channel blockers. Fall-risk medications included benzodiazepine derivative sedatives/hypnotics, zolpidem, opioid analgesics, antipsychotics, antidepressants, and anticonvulsants. Bone-related medications included bisphosphonates (alendronate, risedronate, zoledronic acid), vitamin D, and systemic corticosteroids.

### Propensity score matching

To minimize confounding by indication, we performed 1:1 propensity score matching using a greedy nearest-neighbor algorithm with a caliper width of 0.1 pooled standard deviations [17]. Matching variables included demographics (age at index, sex, race, ethnicity), the comprehensive set of comorbidities including frailty proxies described above, and all concomitant medication classes. Covariate balance was assessed using standardized mean differences (SMD), with values < 0.10 considered indicative of adequate balance.

### Outcomes

#### Primary safety outcomes

Assessed from day 1 through day 365 after the index date included: falls (ICD-10: W00-W19), hip fracture (ICD-10: S72.0-S72.2), acute kidney injury (ICD-10: N17), urinary tract infection (ICD-10: N30.0, N30.9, N39.0), genital candidiasis (ICD-10: B37.3, B37.4), hypoglycemia (ICD-10: E16.0, E16.1, E16.2), volume depletion (ICD-10: E86), hypotension (ICD-10: I95), and syncope (ICD-10: R55).

#### Secondary outcomes

Included: stroke (ICD-10: I63), acute myocardial infarction (ICD-10: I21), heart failure hospitalization (ICD-10: I50), and all-cause mortality.

Patients with outcomes documented prior to the index date were excluded from analysis of that specific outcome to ensure incident events were captured.

### Statistical analysis

Time-to-event analyses were performed using the Kaplan-Meier method. Hazard ratios (HR) with 95% confidence intervals (CI) were estimated using Cox proportional hazards regression comparing event rates between matched cohorts. The proportional hazards assumption was assessed using Schoenfeld residuals. Follow-up extended from day 1 through day 365 after the index date, with censoring applied at loss to follow-up or end of observation period. All analyses were performed within the TriNetX platform.

### Subgroup analyses

To assess the consistency of findings across patient subgroups at high risk for falls, we performed stratified analyses among patients with baseline use of: (1) insulin, (2) benzodiazepine derivative sedatives/hypnotics, and (3) loop diuretics. For each subgroup, both cohorts were restricted to patients with the medication of interest documented within 1 year before the index date, propensity score matching was repeated, and the falls outcome was assessed. Subgroup analyses were reported as cumulative risk ratios rather than hazard ratios to provide a consistent, directly comparable snapshot of event proportions across strata, facilitating assessment of effect direction and magnitude without introducing heterogeneity from differential censoring patterns within each subgroup.

### Sensitivity analysis

To address potential bias from differential mean follow-up duration between arms, we conducted a pre-specified sensitivity analysis censoring all outcomes at 250 days for both cohorts. This fixed landmark was selected as it falls within the observed follow-up range of both arms, ensuring adequate event accrual while eliminating the contribution of differential observation time to outcome ascertainment. Hazard ratios with 95% confidence intervals were estimated using Cox proportional hazards regression, consistent with the primary analysis. Concordance between primary and sensitivity estimates was used to assess the robustness of findings to differential follow-up.

## Results

### Study population, baseline characteristics, and propensity score matching

**Fig. 1:**
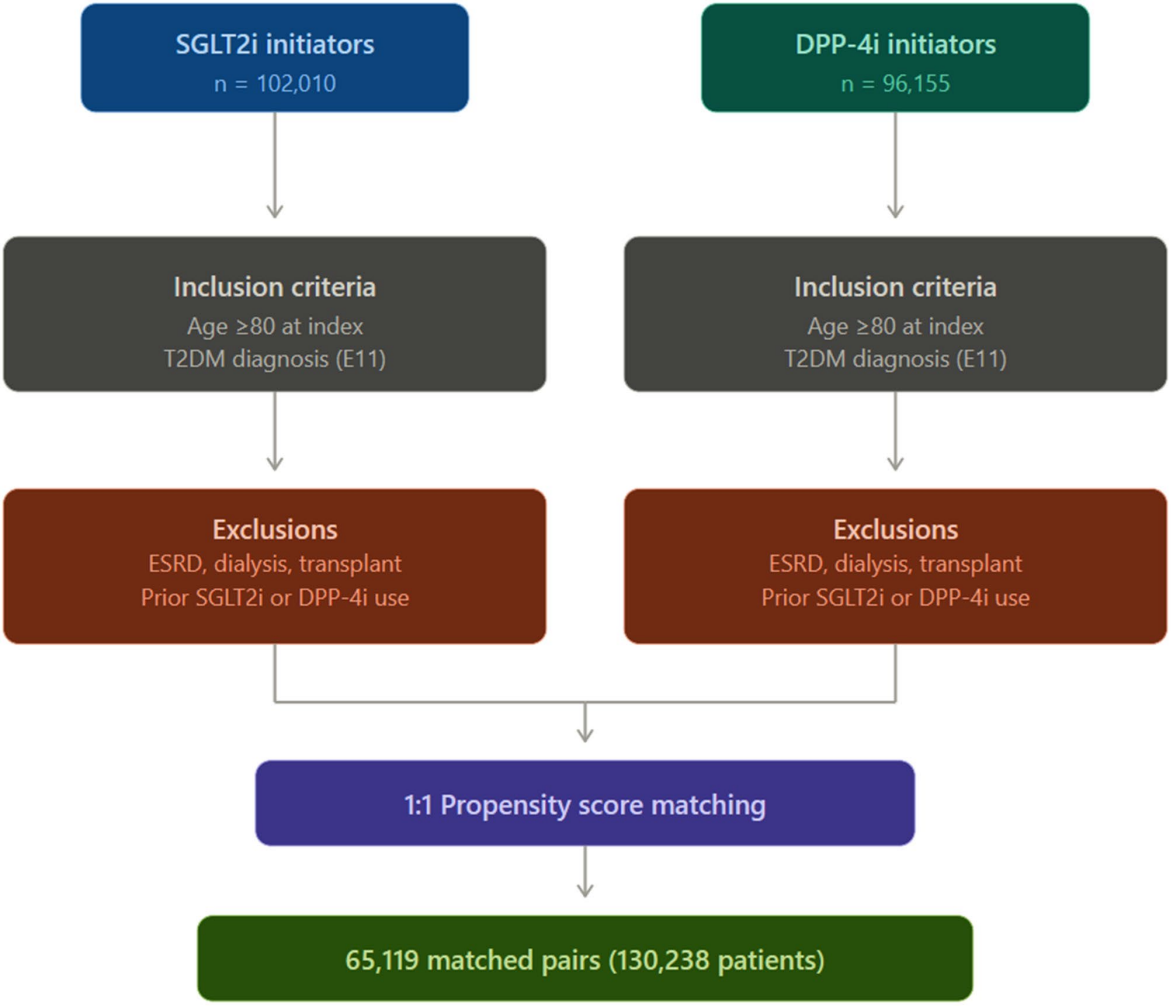
Patient flow diagram showing cohort assembly and propensity score matching. Of 102,010 SGLT2i users and 96,155 DPP-4i users meeting inclusion criteria, 65,119 matched pairs were included in the final analysis

**Fig. 2:**
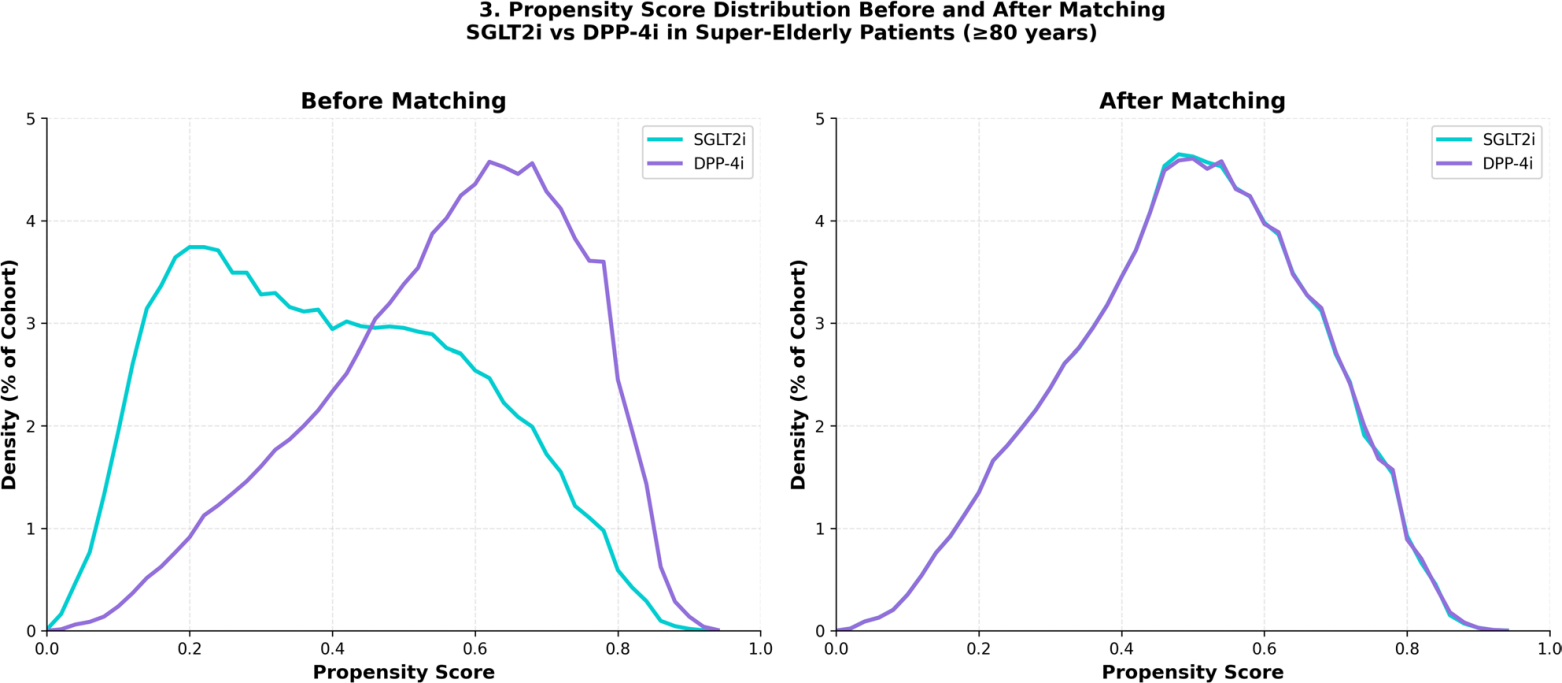
Propensity score density distribution before and after matching. After 1:1 nearest-neighbor matching, the propensity score distributions were well-balanced between SGLT2i and DPP-4i cohorts, demonstrating adequate overlap and covariate balance

Before matching, 102,010 patients were identified in the SGLT2i cohort and 96,155 patients in the DPP-4i cohort. After 1:1 propensity score matching, 65,119 patients remained in each cohort (Fig. 1: Patient Flow Diagram). The propensity score distributions demonstrated excellent overlap after matching (Fig. 2: Propensity Score Distribution before and after match).

The matched cohorts demonstrated excellent covariate balance with all standardized mean differences < 0.02 (Table 1). The mean age at treatment initiation was 82.2 ± 2.3 years in both cohorts (SMD 0.001). Female patients comprised 48.7% of the SGLT2i cohort and 49.1% of the DPP-4i cohort (SMD 0.007). The racial composition was predominantly White (52.7% vs. 52.6%), with Asian patients representing 10.9% and 10.7%, and Black or African American patients representing 9.5% and 9.6%, respectively (all SMD < 0.01).

**Table 1:** Baseline demographics and comorbidities after propensity score matching

Characteristic	SGLT2i (*n* = 65,119)	DPP-4i (*n* = 65,119)	SMD
**Demographics**			
Age at index, years (mean ± SD)	82.2 ± 2.3	82.2 ± 2.4	0.001
Female sex	48.7%	49.1%	0.007
Male sex	51.1%	50.8%	0.006
White	52.7%	52.6%	0.002
Asian	10.9%	10.7%	0.007
Black or African American	9.5%	9.6%	0.004
Hispanic or Latino	4.8%	4.8%	< 0.001
**Cardiovascular Comorbidities**			
Hypertensive diseases	77.0%	76.9%	0.003
Ischemic heart disease	40.8%	41.1%	0.008
Heart failure	24.7%	25.2%	0.013
Atrial fibrillation	22.6%	22.8%	0.006
Cerebral infarction	10.6%	10.7%	0.003
**Metabolic/Renal**			
Type 2 diabetes	80.6%	80.3%	0.008
Chronic kidney disease	34.5%	34.4%	0.002
Overweight/Obesity	19.9%	20.0%	0.003
**Frailty Proxies**			
Unspecified dementia	7.3%	7.4%	0.006
Alzheimer’s disease	2.8%	2.8%	0.006
Vascular dementia	1.8%	1.8%	0.001
Dementia in other diseases	2.9%	3.0%	0.004
Gait and mobility abnormality	11.6%	11.9%	0.009
History of falls	15.1%	15.2%	0.001
Prior femoral fracture	2.9%	3.0%	0.004
Osteoporosis (with fracture)	1.5%	1.5%	0.001
Osteoporosis (without fracture)	11.2%	11.3%	0.003
Malnutrition	3.1%	3.2%	0.007
Cachexia	0.4%	0.4%	0.003
Hypotension	9.3%	9.6%	0.009
Major depressive disorder	2.8%	2.9%	0.003
Parkinson’s disease	2.2%	2.3%	0.003

SMD = standardized mean difference. All SMD < 0.02 indicating excellent balance

The prevalence of key comorbidities after matching was well-balanced: hypertensive diseases (77.0% vs. 76.9%), type 2 diabetes (80.6% vs. 80.3%), ischemic heart disease (40.8% vs. 41.1%), heart failure (24.7% vs. 25.2%), atrial fibrillation (22.6% vs. 22.8%), chronic kidney disease (34.5% vs. 34.4%), and cerebral infarction (10.6% vs. 10.7%; all SMD < 0.02).

Frailty proxy conditions were balanced: unspecified dementia (7.3% vs. 7.4%), Alzheimer’s disease (2.8% vs. 2.8%), other degenerative diseases of the nervous system (4.6% vs. 4.6%), gait and mobility abnormalities (11.6% vs. 11.9%), history of falls (14.8% vs. 15.2%), prior femoral fracture (2.9% vs. 3.0%), osteoporosis with fracture (1.5% vs. 1.5%), osteoporosis without fracture (11.2% vs. 11.3%), malnutrition (3.1% vs. 3.2%), and cachexia (0.4% vs. 0.4%; all SMD < 0.02).

Mean follow-up duration was 258.8 ± 138.2 days in the SGLT2i cohort and 279.2 ± 132.1 days in the DPP-4i cohort, with median follow-up of 365 days in both groups.

### Baseline concomitant medications

All concomitant medication classes were excellently balanced after propensity score matching (Table 2). Beta-blockers were used by 57.1% vs. 57.0% (SMD 0.003), diuretics by 52.5% vs. 52.9% (SMD 0.008), insulin by 46.6% vs. 46.3% (SMD 0.005), metformin by 51.7% vs. 51.2% (SMD 0.010), calcium channel blockers by 47.5% vs. 47.5% (SMD 0.002), angiotensin receptor blockers by 40.9% vs. 41.2% (SMD 0.005), benzodiazepine derivatives by 39.2% vs. 39.6% (SMD 0.009), ACE inhibitors by 33.0% vs. 32.8% (SMD 0.003), anticonvulsants by 24.0% vs. 24.0% (SMD 0.001), antidepressants by 26.7% vs. 26.8% (SMD 0.003), skeletal muscle relaxants by 16.9% vs. 17.2% (SMD 0.007), antipsychotics by 10.6% vs. 10.6% (SMD 0.001), and opioid analgesics by 53.7% vs. 53.5% (SMD 0.002; all SMD ≤ 0.01).

**Table 2:** Baseline concomitant medications after propensity score matching

Medication Class	SGLT2i (*n* = 65,119)	DPP-4i (*n* = 65,119)	SMD
**Diabetes Medications**			
Insulin	46.6%	46.3%	0.005
Metformin	51.7%	51.2%	0.010
Glipizide	16.0%	15.7%	0.008
Glimepiride	14.1%	13.8%	0.007
Glyburide	3.5%	3.6%	0.003
Semaglutide	3.3%	2.9%	0.017
Dulaglutide	3.2%	2.9%	0.019
Liraglutide	1.4%	1.4%	0.002
Exenatide	0.8%	0.7%	0.005
**Cardiovascular Medications**			
Beta-blockers	57.1%	57.0%	0.003
Diuretics (any)	52.5%	52.9%	0.008
Calcium channel blockers	47.5%	47.5%	0.002
Angiotensin receptor blockers	40.9%	41.2%	0.005
ACE inhibitors	33.0%	32.8%	0.003
Anticoagulants	45.9%	46.3%	0.007
Statins	42.6%	42.6%	< 0.001
**Fall-Risk Medications**			
Benzodiazepine derivatives	39.2%	39.6%	0.009
Opioid analgesics	53.7%	53.5%	0.002
Antipsychotics	10.6%	10.6%	0.001
Antidepressants	26.7%	26.8%	0.003
Anticonvulsants	24.0%	24.0%	0.001
Zolpidem	7.3%	7.4%	0.004
**Bone-Related Medications**			
Vitamin D	2.1%	2.1%	< 0.001
Alendronate	4.2%	4.2%	0.001
Zoledronic acid	0.8%	0.8%	0.002
Risedronate	0.4%	0.5%	0.004

SMD = standardized mean difference. All medications assessed in 12 months prior to index date

### Primary safety outcomes

**Fig. 3:**
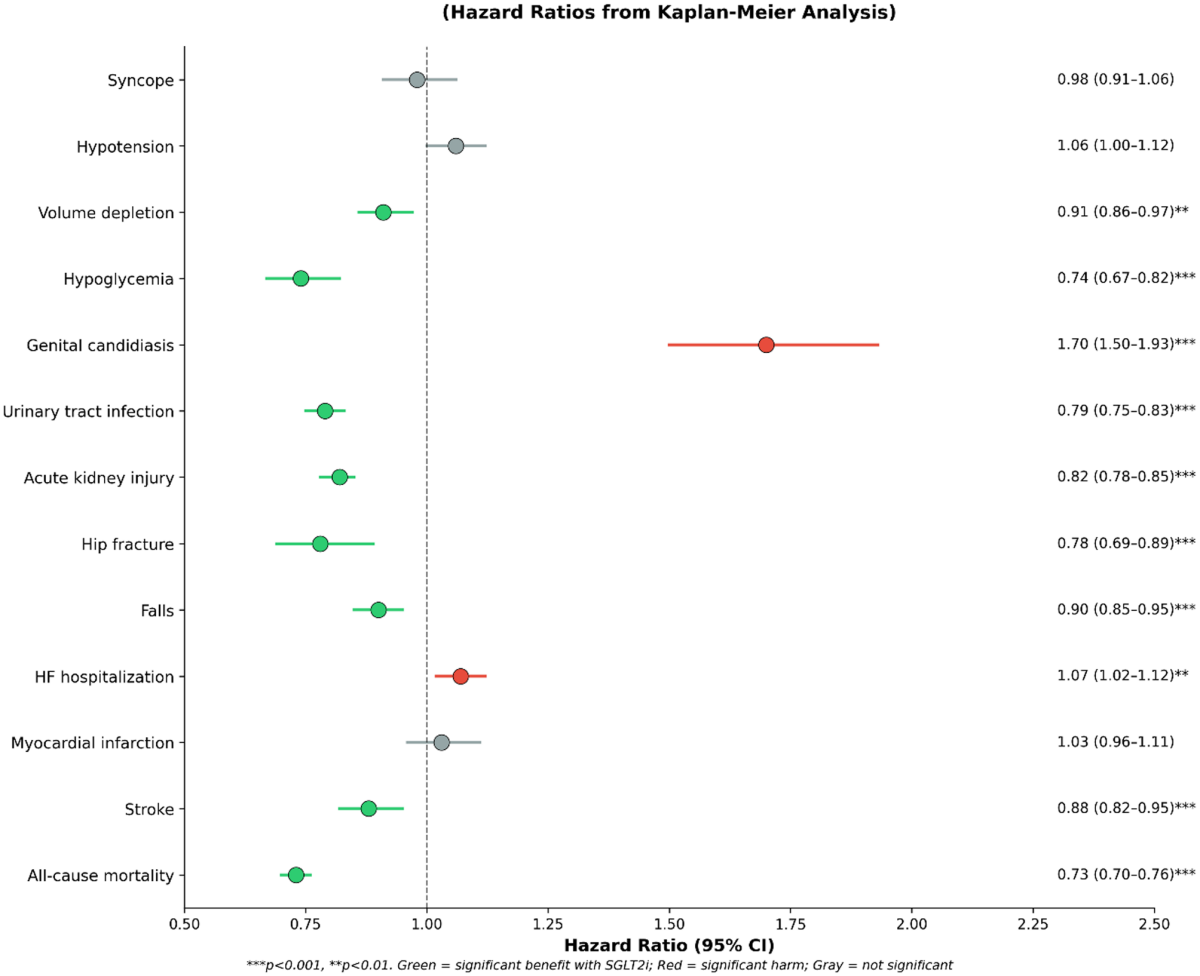
Forest plot of hazard ratios for primary safety and secondary outcomes. SGLT2i was associated with significantly lower risks of falls, hip fracture, acute kidney injury, urinary tract infection, hypoglycemia, volume depletion, stroke, and all-cause mortality compared to DPP-4i. Genital candidiasis and heart failure hospitalization risks were significantly higher with SGLT2i. Hypotension, syncope, and myocardial infarction did not differ significantly between groups. Green indicates significant benefit with SGLT2i; red indicates significant harm; gray indicates no significant difference. HR = hazard ratio; CI = confidence interval. ***p < 0.001, **p < 0.01

Table 3 and Fig. 3 (Primary and Secondary Safety outcomes) summarize hazard ratios for all safety outcomes.

**Table 3:** Primary and secondary outcomes

Outcome	SGLT2i Events/At-Risk (%)	DPP-4i Events/At-Risk (%)	HR (95% CI)	*P*-value
**Primary safety outcomes**				
Falls	2,193/54,644 (4.0%)	2,598/54,471 (4.8%)	0.90 (0.85–0.95)	< 0.001
Hip fracture	418/63,325 (0.66%)	571/63,280 (0.90%)	0.78 (0.69–0.89)	< 0.001
Acute kidney injury	3,219/52,367 (6.1%)	4,057/50,747 (8.0%)	0.82 (0.78–0.85)	< 0.001
Urinary tract infection	3,211/47,358 (6.8%)	4,272/46,913 (9.1%)	0.79 (0.75–0.83)	< 0.001
Genital candidiasis	643/63,889 (1.0%)	404/63,918 (0.6%)	1.70 (1.50–1.93)	< 0.001
Hypoglycemia	654/62,788 (1.0%)	930/62,065 (1.5%)	0.74 (0.67–0.82)	< 0.001
Volume depletion	2,011/58,623 (3.4%)	2,312/57,779 (4.0%)	0.91 (0.86–0.97)	0.002
Hypotension	2,338/58,394 (4.0%)	2,351/58,315 (4.0%)	1.06 (1.00–1.12)	0.06
Syncope	1,300/57,963 (2.2%)	1,417/58,100 (2.4%)	0.98 (0.91–1.06)	0.63
**Secondary outcomes**				
Stroke	1,238/57,570 (2.2%)	1,493/57,502 (2.6%)	0.88 (0.82–0.95)	< 0.001
Myocardial infarction	1,322/57,850 (2.3%)	1,389/58,774 (2.4%)	1.03 (0.96–1.11)	0.44
HF hospitalization	3,231/46,043 (7.0%)	3,257/46,871 (6.9%)	1.07 (1.02–1.12)	0.007
All-cause mortality	3,853/64,778 (5.9%)	5,598/64,722 (8.6%)	0.73 (0.70–0.76)	< 0.001

HR = Hazard Ratio; CI = confidence interval. At-risk populations exclude patients with outcome prior to index date

#### Falls

SGLT2i was associated with significantly lower risk of falls compared to DPP-4i (HR 0.90; 95% CI 0.85–0.95; *p* < 0.001). Among patients without prior falls, 2,193 of 54,644 (4.0%) SGLT2i users and 2,598 of 54,471 (4.8%) DPP-4i users experienced incident falls during follow-up, representing a 10% relative risk reduction.

#### Hip fracture

SGLT2i was associated with significantly lower hip fracture risk (HR 0.78; 95% CI 0.69–0.89; *p* < 0.001). Incident fractures occurred in 418 of 63,325 (0.66%) SGLT2i users versus 571 of 63,280 (0.90%) DPP-4i users, representing a 22% relative risk reduction.

#### Acute kidney injury

SGLT2i was associated with significantly lower AKI risk (HR 0.82; 95% CI 0.78–0.85; *p* < 0.001). AKI occurred in 3,219 of 52,367 (6.1%) SGLT2i users and 4,057 of 50,747 (8.0%) DPP-4i users, representing an 18% relative risk reduction.

#### Urinary tract infection

SGLT2i was associated with significantly lower UTI risk (HR 0.79; 95% CI 0.75–0.83; *p* < 0.001). UTIs occurred in 3,211 of 47,358 (6.8%) SGLT2i users versus 4,272 of 46,913 (9.1%) DPP-4i users, representing a 21% relative risk reduction.

#### Genital candidiasis

SGLT2i was associated with significantly higher genital candidiasis risk (HR 1.70; 95% CI 1.50–1.93; *p* < 0.001). Events occurred in 643 of 63,889 (1.0%) SGLT2i users versus 404 of 63,918 (0.6%) DPP-4i users, representing a 70% relative risk increase.

#### Hypoglycemia

SGLT2i was associated with significantly lower hypoglycemia risk (HR 0.74; 95% CI 0.67–0.82; *p* < 0.001). Events occurred in 654 of 62,788 (1.0%) SGLT2i users versus 930 of 62,065 (1.5%) DPP-4i users, representing a 26% relative risk reduction.

#### Volume depletion

SGLT2i was associated with significantly lower volume depletion risk (HR 0.91; 95% CI 0.86–0.97; *p* = 0.002). Events occurred in 2,011 of 58,623 (3.4%) SGLT2i users versus 2,312 of 57,779 (4.0%) DPP-4i users, representing a 9% relative risk reduction.

#### Hypotension

No significant difference was observed in hypotension risk (HR 1.06; 95% CI 1.00–1.12; *p* = 0.06). Events occurred in 2,338 of 58,394 (4.0%) SGLT2i users and 2,351 of 58,315 (4.0%) DPP-4i users.

#### Syncope

No significant difference was observed in syncope risk (HR 0.98; 95% CI 0.91–1.06; *p* = 0.63). Events occurred in 1,300 of 57,963 (2.2%) SGLT2i users versus 1,417 of 58,100 (2.4%) DPP-4i users.

### Secondary outcomes

#### Stroke

SGLT2i was associated with significantly lower stroke risk (HR 0.88; 95% CI 0.82–0.95; *p* < 0.001). Events occurred in 1,238 of 57,570 (2.2%) SGLT2i users versus 1,493 of 57,502 (2.6%) DPP-4i users, representing a 12% relative risk reduction.

#### Myocardial infarction

No significant difference was observed in MI risk (HR 1.03; 95% CI 0.96–1.11; *p* = 0.44). Events occurred in 1,322 of 57,850 (2.3%) SGLT2i users and 1,389 of 58,774 (2.4%) DPP-4i users.

#### Heart failure hospitalization

SGLT2i was associated with modestly higher HF hospitalization risk (HR 1.07; 95% CI 1.02–1.12; *p* = 0.007). Events occurred in 3,231 of 46,043 (7.0%) SGLT2i users and 3,257 of 46,871 (6.9%) DPP-4i users, representing a 7% relative risk increase.

#### All-cause mortality

SGLT2i was associated with dramatically lower all-cause mortality (HR 0.73; 95% CI 0.70–0.76; *p* < 0.001). Deaths occurred in 3,853 of 64,778 (5.9%) SGLT2i users versus 5,598 of 64,722 (8.6%) DPP-4i users, representing a 27% relative risk reduction.

### Subgroup analyses: falls by concomitant medication use

**Fig. 4:**
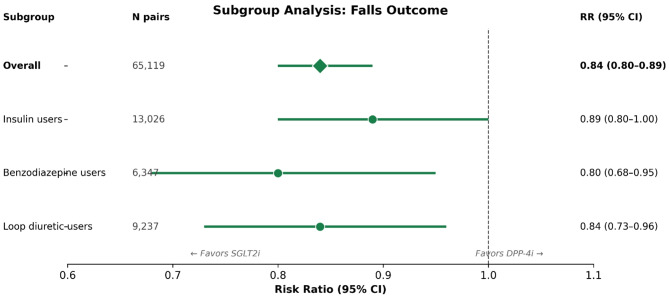
Subgroup analysis forest plot showing falls risk ratio by concomitant medication use. The protective effect of SGLT2i on falls was consistent across subgroups using insulin (RR 0.89), benzodiazepines (RR 0.80), and loop diuretics (RR 0.84). Diamond represents overall estimate; circles represent subgroup estimates with size proportional to sample size. RR= Risk Ratio

Subgroup analyses were reported as cumulative risk ratios rather than hazard ratios to provide a consistent, directly comparable snapshot of event direction across groups. The protective effect of SGLT2i on falls was consistent across subgroups defined by baseline use of medications known to affect fall risk (Table 4; Fig. 4: Subgroup Analysis):

**Table 4:** Subgroup analysis: falls outcome by concomitant medication use

Subgroup	*N* Pairs	SGLT2i Falls (%)	DPP-4i Falls (%)	RR (95% CI)	*P*-value
Overall	65,119	2,193/54,644 (4.0%)	2,598/54,471 (4.8%)	0.84 (0.80–0.89)	< 0.001
Insulin users	13,026	532/9,957 (5.3%)	595/9,929 (6.0%)	0.89 (0.80–1.00)	0.048
Benzodiazepine users	6,347	237/5,061 (4.7%)	293/5,024 (5.8%)	0.80 (0.68–0.95)	0.010
Loop diuretic users	9,237	365/7,347 (5.0%)	434/7,299 (5.9%)	0.84 (0.73–0.96)	0.009

All subgroups showed significant or borderline significant fall protection with SGLT2i. Consistent direction of the cumulative Risk Ratio (rather than the primary Hazard Ratio) across subgroups indicates no evidence of effect modification. RR= Risk Ratio

#### Insulin users (*n* = 13,026 matched pairs)

Falls occurred in 532 of 9,957 (5.3%) SGLT2i users versus 595 of 9,929 (6.0%) DPP-4i users (RR 0.89; 95% CI 0.80–1.00; *p* = 0.048).

#### Benzodiazepine users (*n* = 6,347 matched pairs)

Falls occurred in 237 of 5,061 (4.7%) SGLT2i users versus 293 of 5,024 (5.8%) DPP-4i users (RR 0.80; 95% CI 0.68–0.95; *p* = 0.010).

#### Loop diuretic users (*n* = 9,237 matched pairs)

Falls occurred in 365 of 7,347 (5.0%) SGLT2i users versus 434 of 7,299 (5.9%) DPP-4i users (RR 0.84; 95% CI 0.73–0.96; *p* = 0.009).

The direction and magnitude of the fall’s reduction was consistent across all subgroups, with no evidence of significant effect modification.

### Sensitivity analysis

To evaluate the robustness of primary and secondary findings to differential follow-up duration between arms (mean 259 days SGLT2i vs. 279 days DPP-4i), a sensitivity analysis was conducted censoring all outcomes at a fixed 250-day landmark. Results were highly consistent with the primary analysis across all outcomes (Supplementary Table S1). The falls risk reduction persisted (HR 0.89; 95% CI 0.84–0.95), as did reductions in hip fracture (HR 0.77; 0.66–0.89), as well as acute kidney injury, urinary tract infection, hypoglycemia, stroke, and all-cause mortality. Volume depletion remained significantly reduced (HR 0.91; 0.85–0.98; *p* = 0.010). Genital candidiasis risk remained elevated with SGLT2i and heart failure hospitalization remained modestly increased, consistent with primary estimates. Hypotension, syncope, and myocardial infarction remained non-significant. The near-identical estimates across all primary and secondary analyses confirm that differential follow-up duration does not materially influence the observed associations.

## Discussion

### Principal findings

In this large propensity score-matched cohort study of 130,238 super-elderly patients (≥ 80 years) with type 2 diabetes initiating treatment with either SGLT2i or DPP-4i, we found that SGLT2i demonstrated a remarkably favorable safety profile compared to DPP-4i. SGLT2i use was associated with 10% lower fall risk, 22% fewer hip fractures, 18% less acute kidney injury, 21% fewer urinary tract infections, 26% less hypoglycemia, 9% less volume depletion, 12% fewer strokes, and 27% lower all-cause mortality. These benefits persisted across subgroups stratified by concomitant use of insulin, benzodiazepines, and loop diuretics. Importantly, all assessed concomitant medications, including fall-risk, hypoglycemia-associated, and volume-depleting agents, were well balanced after matching (all SMD ≤ 0.01), strengthening confidence that these findings are not driven by treatment-related confounding. Similarly, multiple frailty-related conditions were balanced between groups (all SMD < 0.02), reducing the likelihood of channeling bias, although residual confounding from unmeasured frailty components cannot be entirely excluded. The only safety concern was an expected 70% increase in genital candidiasis, a well-characterized class effect of SGLT2i, while hypotension risk was not increased despite theoretical concerns about volume depletion in this age group. Present study specifically targets patients ≥ 80 years at treatment initiation (mean age 82 years) with a larger sample size (65,119 matched pairs) and demonstrates benefits across broad range of safety outcomes.

### Falls and fracture protection

The 10% reduction in falls and 22% reduction in hip fractures observed with SGLT2i compared to DPP-4i represents a clinically significant finding with important implications for super-elderly patients. Falls are the leading cause of injury-related morbidity and mortality in older adults, and hip fractures are associated with substantial functional decline, institutionalization, and death [18]. Our findings are consistent with a Medicare study demonstrating 18% lower fall risk with SGLT2i versus DPP-4i in patients aged ≥ 66 years [19], and extend these findings to the super-elderly population. Present finding of significantly reduced hip fracture risk directly addresses concerns raised by CREDENCE trial, which reported a fracture signal (HR 0.98; 95% CI 0.70–1.37) [20]. Importantly, this signal was not replicated in the CANVAS Program (HR 1.26; 95% CI 1.04–1.52), despite using the same agent (canagliflozin) in a higher-risk population with chronic kidney disease [6]. A comprehensive meta-analysis of 20 randomized controlled trials including 12,764 patients found no significant association between SGLT2i use and elevated fracture risk (pooled RR 1.21; 95% CI 0.95–1.54), with no substantial effects on bone mineral density or bone metabolism markers [21]. Expert interpretation from the CANVAS/CREDENCE investigators concluded that the initial fracture signal was likely a chance observation rather than a true class effect [22].

Several mechanisms may explain the fall and fracture protection observed with SGLT2i. First, the 26% reduction in hypoglycemia with SGLT2i compared to DPP-4i likely contributes, as hypoglycemia is a well-established risk factor for falls in elderly diabetic patients [23]. Second, SGLT2i have been associated with improvements in body composition, including preservation of lean muscle mass relative to fat mass loss, which may improve strength and balance [24]. Third, emerging evidence suggests SGLT2i may have direct effects on bone metabolism, though the mechanisms remain incompletely understood [25].

The protective effect of SGLT2i on falls persisted across subgroups of patients using insulin (11% reduction), benzodiazepines (20% reduction), and loop diuretics (16% reduction). This finding is particularly reassuring, as these medications are commonly prescribed in elderly patients and are each associated with increased fall risk through distinct mechanisms: hypoglycemia (insulin), sedation (benzodiazepines), and volume depletion/orthostatic hypotension (loop diuretics). The consistency of benefit across these high-risk subgroups suggests that SGLT2i provides additive protection against falls regardless of other fall-risk medications.

### Renal safety

Contrary to early concerns about SGLT2i-induced acute kidney injury due to volume depletion and hemodynamic effects, we observed a 18% lower risk of AKI with SGLT2i compared to DPP-4i. This finding is consistent with the nephroprotective effects demonstrated in clinical trials including CREDENCE and DAPA-CKD [20, 26], and extends these benefits to super-elderly patients who were underrepresented in those trials. Furthermore, meta-Analysis Cardio-Renal Trialists’ Consortium, pooling data from 13 large placebo-controlled trials with over 90,000 participants, demonstrated that SGLT2i reduced AKI risk by 23% [27]. The mechanism likely involves SGLT2i-mediated reduction in intraglomerular pressure through tubuleglomerular feedback, decreased hyperfiltration, and anti-inflammatory effects [28].

### Volume-related events

We observed a 9% reduction in volume depletion events and no increase in hypotension compared to DPP-4i. This counterintuitive finding may be explained by the osmotic diuresis induced by SGLT2i being more gradual than that of loop diuretics, allowing for compensatory mechanisms to maintain blood pressure. Additionally, the reduction in heart failure-related congestion may offset any volume-depleting effects. Despite the diuretic mechanism of SGLT2i, current ADA guidelines recommend cautious use of SGLT2i medications in older frail patients due to the risk of clinically significant volume depletion [29].

### Mortality benefit

The 27% reduction in all-cause mortality observed with SGLT2i compared to DPP4i is striking and likely reflects the cumulative benefits across multiple organ systems. Cardiovascular mortality reduction has been consistently demonstrated in SGLT2i trials [6, 30, 31], and our observation of 12% lower stroke risk that may contributes to this benefit. The reductions in falls, fractures, AKI, and hypoglycemia each associated with mortality in elderly patients likely provide additional survival benefit in this population. Though the reduction in all-cause mortality for both groups warrants cautious interpretation, as it may partly reflect relative safety benefit, the diverse population with potentially different baseline cardiovascular risk profiles, limited follow-up period, healthcare utilization patterns, and SGLT2i and DPP-4i prescribing thresholds.

### Genital candidiasis

The 70% increase in genital candidiasis with SGLT2i compared to DPP-4i is expected given the mechanism of glucosuria and is consistent across all SGLT2i trials [32]. While this adverse effect is generally mild and treatable, it may be particularly bothersome for some elderly patients. Appropriate patient counseling and hygiene recommendations can mitigate this risk.

Genital candidiasis rates (1.0% vs. 0.6%) were lower than prospective trials and infection-specific retrospective cohorts [33], likely reflecting under-ascertainment in administrative EHR data due to self-treated infections, elderly underreporting, and absence of protocol-driven screening. Despite this, the 70% relative increase with SGLT2i remained significant and consistent with the known class effect.

### Clinical implications

These findings have important implications for clinical practice. Current prescribing information for some SGLT2i cautions against initiation in patients aged ≥ 85 years due to limited therapeutic experience. Our data from over 65,000 matched super-elderly patients suggest that, rather than being contraindicated, SGLT2i may offer substantial safety advantages over DPP-4i in this population. The benefits in falls, fractures, AKI, hypoglycemia, and mortality—outcomes of particular importance in geriatric care—support consideration of SGLT2i in appropriately selected super-elderly patients with type 2 diabetes.

Clinicians should weigh the favourable safety profile demonstrated here against the expected increase in genital candidiasis and the modest increase in heart failure hospitalization. The latter finding is paradoxical given SGLT2i’s established cardioprotective effects and may reflect confounding by indication or surveillance bias rather than a true adverse effect. Patient selection should consider individual risk factors, with particular attention to patients who may be at high risk for genital infections or decompensated heart failure.

### Study limitations

Several limitations warrant consideration. First, as an observational study using a global administrative data, residual confounding remains possible – such as prescription patterns and healthcare utilization patterns – despite rigorous propensity score matching. Second, laboratory values including HbA1c, eGFR, and BMI are inconsistently captured in TriNetX; we used diagnosis codes (CKD stages, obesity) as proxies. Third, treatment discontinuation and switching could not be directly assessed; however, the consistency of benefits across outcomes suggests findings are robust. Fourth, this is a comparison of SGLT2i versus DPP-4i specifically; findings should not be interpreted as demonstrating absolute safety but rather relative safety compared to this active comparator. Fifth, while we matched on frailty proxies, validated frailty instruments are not available in electronic health records. Sixth, mean follow-up differed slightly between groups (259 vs. 279 days); however, sensitivity analysis with fixed 250-day follow-up confirmed all primary findings, indicating that differential follow-up does not explain the observed associations. The modestly increased heart failure hospitalization with SGLT2i (HR 1.07) was consistent across primary and sensitivity analyses, though unexpectedly paradoxical given known cardioprotective effects of SGLT2i in clinical trials; this finding may reflect residual confounding or differences in HF severity at baseline warranting further investigation. Finally, the median follow-up period in our study may be insufficient to detect differences in some hard endpoints, such as cardiovascular outcomes and mortality, as these typically require longer durations of exposure.

## Conclusions

In super-elderly patients (≥ 80 years) with type 2 diabetes, SGLT2 inhibitors demonstrated a generally favourable safety profile compared to DPP-4 inhibitors. SGLT2i was associated with significantly lower risks of falls (10% reduction), hip fracture (22% reduction), acute kidney injury (18% reduction), urinary tract infection (21% reduction), hypoglycemia (26% reduction), volume depletion (9% reduction), stroke (12% reduction), and all-cause mortality (27% reduction). The falls reduction was consistent across subgroups using insulin, benzodiazepines, and loop diuretics. Safety concerns included increased genital candidiasis (70% increase) and modestly higher heart failure hospitalization (7% increase). These findings support the use of SGLT2 inhibitors in appropriately selected super-elderly patients and should inform shared decision-making in this vulnerable and rapidly growing population.

## Supplementary Information

Below is the link to the electronic supplementary material.

Supplementary Material 1

## Data Availability

The data used in this study are available through the TriNetX platform and are subject to data use agreements with participating healthcare organizations. Individual-level patient data cannot be shared publicly. Aggregated outputs and analytic queries generated within TriNetX can be made available from the corresponding author upon reasonable request, subject to TriNetX policies.

## Abbreviations

T2DM: Type 2 diabetes mellitus
SGLT2i: Sodium—glucose cotransporter—2 inhibitors
DPP-4i: Dipeptidyl peptidase—4 inhibitors
AKI: Acute kidney injury
CKD: Chronic kidney disease
ESRD: End—stage renal disease
GLP-1: Glucagon—like peptide—1
ACE: Angiotensin—converting enzyme
UTI: Urinary tract infection
CPT: Current Procedural Terminology
ICD: International Classification of Diseases
HIPAA: Health Insurance Portability and Accountability Act
ADA: American Diabetes Association
HR: Hazard ratio
RR: Risk ratio
CI: Confidence interval
SMD: Standardized mean difference
