# Supplementary material for: Safety of SGLT2 inhibitors versus DPP-4 inhibitors in super-elderly patients (≥ 80 years) with type 2 diabetes: a propensity score-matched cohort study
Source: Cardiovasc Diabetol Endocrinol Rep. 2026 Jun 29;12:35. doi: 10.1186/s40842-026-00305-4 (PMC13312745; doi:10.1186/s40842-026-00305-4)
Supplement: Supplementary file 1 — Supplementary Material 1 [file 40842_2026_305_MOESM1_ESM.docx]

**SUPPLEMENTARY MATERIALS**

**Supplementary Table S1.** Sensitivity Analysis with Fixed 250-Day Follow-up

| Outcome | Primary Analysis HR (95% CI) | Sensitivity Analysis HR (95% CI) | P-value |
| --- | --- | --- | --- |
| Falls | 0.90 (0.85–0.95) | 0.89 (0.84–0.95) | <0.001 |
| Hip fracture | 0.78 (0.69–0.89) | 0.77 (0.66–0.89) | <0.001 |
| Acute kidney injury | 0.82 (0.78–0.85) | 0.82 (0.78–0.86) | <0.001 |
| Urinary tract infection | 0.79 (0.75–0.83) | 0.77 (0.73–0.81) | <0.001 |
| Genital candidiasis | 1.70 (1.50–1.93) | 1.82 (1.57–2.10) | <0.001 |
| Hypoglycemia | 0.74 (0.67–0.82) | 0.69 (0.61–0.78) | <0.001 |
| Volume depletion | 0.91 (0.86–0.97) | 0.91 (0.85–0.98) | 0.010 |
| Hypotension | 1.06 (1.00–1.12) | 1.03 (0.97–1.10) | 0.332 |
| Syncope | 0.98 (0.91–1.06) | 0.96 (0.88–1.05) | 0.381 |
| Stroke | 0.88 (0.82–0.95) | 0.85 (0.78–0.93) | <0.001 |
| Myocardial infarction | 1.03 (0.96–1.11) | 1.05 (0.96–1.15) | 0.256 |
| HF hospitalization | 1.07 (1.02–1.12) | 1.11 (1.04–1.17) | <0.001 |
| All-cause mortality | 0.73 (0.70–0.76) | 0.73 (0.69–0.76) | <0.001 |

**Note:** Sensitivity analysis censored all outcomes at 250 days to address differential mean follow-up duration (259 days SGLT2i vs 279 days DPP-4i). HR = hazard ratio.
